# The e-STROKE Study: The Design of a Prospective Observational Multicentral Study

**DOI:** 10.3390/jcdd12010017

**Published:** 2025-01-03

**Authors:** Kateřina Dvorníková, Veronika Kunešová, Svatopluk Ostrý, Robert Mikulík, Michal Bar

**Affiliations:** 1 Department of Neurology, University Hospital in Ostrava, 70800 Ostrava, Czech Republic; katerina.dvornikova@fno.cz; 2 Cerebrovascular Research Program, International Clinical Research Center, 65691 Brno, Czech Republic; veronika.kunesova@fnusa.cz (V.K.); mikulik@hotmail.com (R.M.); 3 Department of Imaging Methods, Faculty of Medicine, Ostrava University, 70103 Ostrava, Czech Republic; 4 Neurology Department, Regional Hospital České Budějovice, 37001 České Budějovice, Czech Republic; ostry.svatopluk@nemcb.cz; 5 Neurology Department, T. Baťa Regional Hospital Zlín, 76275 Zlín, Czech Republic

**Keywords:** stroke, CT imaging, multimodal CT, predictive value, CT perfusion, stroke mimics

## Abstract

**Introduction:** The e-STROKE study is a prospective, multicenter observational study designed to assess the impact of various CT parameters (including e-ASPECT, CT perfusion (CTP), collateral flow status, and the size and location of the ischemic lesion) on the clinical outcomes of patients with ischemic stroke, as evaluated by the modified Rankins Scale (mRS) three months post-stroke. This study also aims to investigate whether the use of multimodal CT imaging increases the number of patients eligible for recanalization therapy. The analysis will integrate data from the RES-Q registry and radiological data from the e-STROKE system provided by Brainomix Ltd. **Aims:** The primary aim is to determine the predictive value of CT parameters (e-ASPECTS, CTP, collateral vessel status, and ischemic lesion volume and location) on three-month functional outcomes, as defined by the mRS, in patients with non-lacunar stroke following recanalization treatment (IVT and/or MT). The secondary aim is to evaluate whether multimodal CT examination leads to an increase in the number of patients eligible for recanalization therapy. Additionally, this study seeks to assess the specificity and sensitivity of multimodal CT in distinguishing stroke mimics from actual strokes. **Methods:** This multicenter observational study involves patients with suspected acute ischemic stroke and a premorbid mRS ≤ 4, who are treated with endovascular thrombectomy (EVT), intravenous thrombolysis (IVT), or managed conservatively in stroke centers within the Czech Stroke Research Network (STROCZECH), which is part of the Czech Clinical Research Infrastructure Network (CZECRIN). Data collection includes demographic, clinical, and imaging data variables such as age, sex, ethnicity, risk factors, treatment times (OTT, DNT, and OGT), TICI scores, post-treatment hemorrhage (ECAS II), mRS outcome, stroke etiology, e-ASPECTS, acute ischemic volume (AIV), thrombus length on NCCT, CTA collateral score and collateral vessel density, location of large vessel occlusion, ischemic core, hypoperfusion volume, mismatch ratio and volume, final infarct volume, hemorrhage volume, and MRI in case of negative follow-up NCCT. **Conclusions:** We anticipate collecting robust clinical and radiological data from approximately 2000 patients across 22 centers over a 12-month period. The results are expected to enhance the precision of diagnostic and prognostic radiological markers in managing acute stroke.

## 1. Introduction

Baseline and follow-up diagnostic neuroimaging are critical components in stroke diagnosis, management, treatment, and outcome prediction. Currently, the patient’s selection for interventions is guided by two main factors: clinical characteristics (e.g., the severity of neurological deficit, premorbid functional status, and the time of symptom onset) and brain imaging. Brain computed tomography (CT) is the most widely used imaging modality in stroke care; non-contrast CT (NCCT), CT angiography (CTA), and CT perfusion (CTP) are essential tools that complement clinical examination and patient history, aiding in diagnosis and therapeutic decision-making.

The Alberta Stroke Program Early CT Score (ASPECTS) is the most validated and widely used score for assessing early ischemic changes in the anterior circulation. It is a critical imaging selection tool for endovascular thrombectomy (EVT) and has been shown to significantly predict functional outcomes [1,2,3]. However, while it represents a validated grading system, its inter-rater reliability is limited. There is an ongoing effort to develop reliable and accurate software tools to assist stroke physicians with scan assessment and decision-making.

Neuroimaging interpretation in acute stroke requires significant expertise, potentially leading to delays among less experienced physicians. Therefore, stroke clinicians require simple, quick, and accurate imaging tools. The e-ASPECTS software (Brainomix, Oxford, UK) offers a fully automated ASPECT scoring tool for NCCT, which has demonstrated scoring at an expert level in a previous study [4,5,6,7,8]. The e-ASPECTS has the potential to reduce inter-rater variability and has been utilized in patient selection for randomized clinical trials [9]. In addition, RAPID software, which automates post-processing, has been employed as an accurate predictor of infarct score and penumbra using CTP or MRI in clinical trials.

Baseline neuroimaging also plays a crucial role in determining the extent of irreversibly damaged brain tissue, as extensive early ischemic changes decrease the likelihood of tissue salvage through EVT and increase the risk of reperfusion injury and futile recanalization. CTP and multiphase CTA (mCTA) are the most commonly used imaging techniques for assessing salvageable tissue at risk and have proven effective in EVT patient selection during randomized controlled trials. Each technique has its pros and cons: CTP maps are straightforward and visually intuitive, even for less experienced clinicians, while recent evidence suggests that a positive CTP (indicating hypoperfusion) strongly predicts ischemic stroke, warranting comprehensive stroke care without delay [10,11,12,13,14].

In recent years, individual CTP parameters (e.g., ischemic core volume estimate, hypoperfusion volume, and mismatch ratio/volume) have gained attention for their potential utility not only in guiding treatment but also as biomarkers for predicting clinical outcomes. For example, EVT has improved clinical outcomes across a wide range of ischemic core volumes and penumbral profiles, even in patients with an ASPECTS of 3 and an ischemic core of 150 mL or larger [15].

Hemorrhagic transformation (HT) or parenchymal hematoma (PH) are complications associated with both IVT and MT. Various clinical scores, such as SEDAN [16], have been developed to predict the risk of intracerebral hemorrhage (ICH), although their clinical application remains limited [17]. One potential explanation for HT/PH occurrence is the depth of ischemia, which can be approximated from baseline CTP studies. Predictors of HT/PH, including infarct volume [18], significantly delayed cerebral blood flow (CBF) [19]; low cerebral blood volume (CBV) [20] can be derived from CTP analysis. A meta-analysis by Chong et al. evaluated 15 studies on the predictive value of CTP for HT, encompassing 1134 patients. Their findings indicated that high blood–brain barrier permeability and hypoperfusion status, as derived from CTP, were associated with HT [21].

Follow-up NCCT or MRI performed more than 24 h after recanalization treatment are both clinically utilized to exclude hemorrhagic complications and to visually estimate the extent and location of the ischemic lesion [22,23,24,25,26]. Hypodensity (hypoattenuation) on follow-up NCCT and hyperintensity on diffusion-weighted imaging (DWI) MRI are believed to represent irreversibly injured tissue, including cytotoxic and vasogenic edema. DWI, in particular, is a highly sensitive diagnostic modality for detecting acute ischemic lesions in stroke patients [27]. Previous studies have shown that the ischemic lesion volume (ILV), defined as the volume of brain infarction/acute ischemia measured on follow-up scans more than 24 h post-treatment, correlates with the 3-month functional outcomes defined by the mRS [22,24,26]. However, evidence supporting this correlation remains limited. The localization of brain infarction on follow-up CT or MRI is needed for setting of etiology of the stroke (lacunar versus embolic).

Despite the potential value of early prediction based on ILV, routine clinical practice does not typically include ILV measurement due to the time demands of manual volumetric measurements and limited personnel resources. These limitations might be overcome by reliable automatic software. A high degree of agreement has been found between ILVs measured by automated software (for NCCT) and manual outlining (for DWI-MRI) on follow-up stroke neuroimaging in patients who underwent MT for large vessel occlusion (LVO) in the anterior cerebral circulation [28].

**Study questions:**

1. **Baseline imaging:** To assess the association between baseline automated total e-ASPECTS and regional e-ASPECT with 3-month mRS outcomes.
2. **Baseline imaging:** To evaluate the association between baseline automated e-ASPECT and infarct volume size 24–36 h after recanalization therapy.
3. **Baseline imaging:** To analyze the correlation between baseline CTP and infarct volume size 24–36 h after recanalization therapy.
4. **Baseline imaging:** To determine the prognostic value of CTP automated core and mismatch measurement and 3-month mRS outcomes.
5. **Baseline imaging:** To examine the diagnostic accuracy of CTP in predicting parenchymal hematoma type I and II, as defined by ECASS criteria, in patients treated with tPA and/or EVT.
6. **Follow-up imaging (CT or MRI):** To investigate the relationship between follow-up (24–36 h) automated ILV measurement with the 3-month functional outcome as defined by mRS.
7. **Baseline imaging:** To assess whether the introduction of CTP in routine stroke care increases the number of procedures (IVT and MT).
8. **Baseline imaging:** To compare the diagnostic capability of multimodal CT imaging (NCCT, CTA, and CTP) with DWI MRI to distinguish stroke mimics from true ischemic stroke.

## 2. Materials and Methods

The e-STROKE study is a multicenter observational study involving patients with acute ischemic non-lacunar stroke who are treated with EVT, IVT, or conservatively, in stroke centers within the Czech Stroke Research Network (STROCZECH, Brno, Czech Republic), part of the Czech Clinical Research Infrastructure Network (CZECRIN, Brno, Czech Republic).

**Inclusion criteria:**

- Patients with acute ischemic stroke within 24 h of onset who are treated with IVT, and/or MT, or conservatively.
- Patients with an unknown time of stroke onset.
- Patients with wake-up stroke (i.e., stroke symptoms presenting upon waking).
- Age ≥ 18 years.
- Signed informed consent.

**Exclusion criteria:**

- Modified Rankin Scale (mRS) score > 4 prior to the stroke event.
- Presence of acute hemorrhage or other findings on NCCT that exclude the diagnosis of ischemic stroke.

**Collected Demographic and Clinical Data:**

- Age;
- Sex;
- Ethnicity;
- Risk factors;
- Time to treatment (Onset to Treatment [OTT], Door to Needle Time [DNT], Onset to Groin Time [OGT]);
- Thrombolysis in Cerebral Infarction (TICI) score;
- Presence of hemorrhage after IVT or MT (as per ECASS II criteria);
- Clinical outcome in mRS;
- Stroke etiology.

**Imaging protocol/s:** All patients with suspected acute stroke will undergo routine baseline NCCT and single-phase CTA from the aortic arch to the vertex. Follow-up neuroimaging includes NCCT (standard of care in the Czech Republic) conducted 24 to 36 h after EVT or IVT treatment. The timing of follow-up imaging is >24 h post-treatment, as this represents the earliest time point for accurately delineating the acute ischemia volume [7].

- **NCCT** will be performed on a multi-detector spiral 64-series CT scanner. The NCCT examination is followed by CTA using 50–100 mL of iodine contrast agent (Visipaque, GE Healthcare, Piscataway, NJ, USA) administered at a rate of 4 mL/s. The CTA will cover the aortic arch to the distal intracranial arteries. The basic CT section width for further reconstruction is 0.75 mm.
- The automated processing of NCCT, CTA, and CTP will be performed using the latest CE-marked 11.5. version of e-Stroke software Brainomix 360 (Brainomix, Oxford, UK) at baseline. Follow-up imaging will be processed using algorithms currently in development by Brainomix. The e-Stroke image processing algorithms employ an AI approach, combining traditional 3D graphics with statistical methods and machine learning classification techniques. These algorithms have been trained on a large dataset (>10,000 images) of real-world CT scans from stroke patients and negative controls, with ground-truth data from additional imaging modalities, such as MRI, acquired within 1–2 h of the CT scan, along with other modalities and clinical information. This dataset includes CT scans from all major scanner manufacturers collected from a wide range of countries worldwide.
- Within e-Stroke, NCCT, CTA, and CTP will be processed using the e-ASPECTS [4,7,28,29], e-CTA [7,8], and e-CTP modules, respectively. It is important to note that e-Stroke is intended to be used as a decision-support tool, with results designed to be interpreted within the clinical context by the user.
- **MRI Examination** will be conducted on a Siemens Prisma, 3.0 T (Siemens, Erlangen, Germany) scanner. The imaging protocol includes the following:
  ◦ Localizers;
  ◦ Diffusion-weighted imaging (DWI) with b-factor values of 0, 400, and 800 s/mm^2^, calculated b-value of 1000 s/mm^2^, slice thickness (ST) of 3.0 mm, GAP of 0.6 mm, repetition time (TR) of 2900 ms, echo time (TE) of 60 ms, field of view (FOV) of 256 mm, bandwidth (BW) of 868 Hz/Px, 40 slices, and an acquisition time (TA) of 2:09;
  ◦ Reconstructed apparent diffusion coefficient (ADC) maps;
  ◦ 3D FLAIR sequence, performed in the sagittal plane with 1 mm multi-planar reconstruction (MPR) in axial and coronal planes, TR 5500 ms, TE 383 ms, ST 1 mm, 160 slices, GAP 0, inversion time (TI) 1800 ms, flip angle (FA) T2 Variable degrees, BW 751 Hz/Px, and TA 3:46. The total examination time is 6:12 plus shimming time.

1. **Baseline Imaging**:
  - NCCT:
    ◦ e-ASPECTS;
    ◦ e-ASPECTS acute ischemic volume (AIV);
    ◦ Thrombus length on NCCT (if identified).
  - CTA:
    ◦ CTA collateral score (CTA-CS, also known as Tan [30,31] score);
    ◦ CTA collateral vessel density;
    ◦ Location of large vessel occlusion (proximal versus distal, if identified).
  - CTP:
    ◦ Ischemic core volume estimate, defined as relative CBF < 30%;
    ◦ Hypoperfusion volume estimate, defined as Tmax > 6 s;
    ◦ Mismatch ratio;
    ◦ Mismatch volume;
    ◦ Alternative estimate of ischemic core volume using rCBF threshold of <38%;
    ◦ Hypoperfusion intensity ratio.
2. **Follow-up**
  - 24–36 h NCCT:
    ◦ Final infarct volume (FIV);
    ◦ Hemorrhage volume (if any).
  - MRI examination in patients with a negative follow-up brain NCCT.

**Figure 1:**
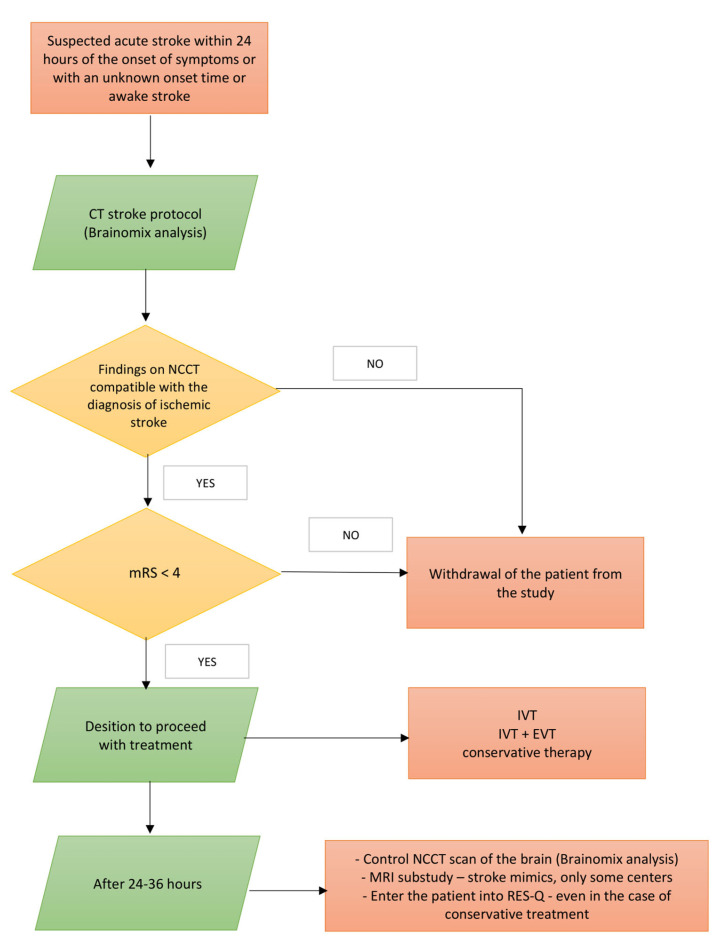
Entry of subjects into the study.

CT and MRI parameters will be evaluated independently by a central neuroimaging laboratory. For the trial flow, refer to the diagram depicting the entry of subjects into the study (Figure 1).

**Statistical analysis:** We anticipate achieving a successful recanalization rate of approximately 70% across the entire cohort. Based on prior studies in the literature, the overall probability of obtaining a good clinical outcome is expected to be 40–50%.

The required sample size for this type of study is computed using Demedenko’s method and a logistic regression model, powering the study to 90% to detect a minimum adjusted odds ratio of 2.0 for good clinical outcomes across the population. The sample size ranges from 270 to 496, depending on recanalization estimates ranging from 70% to as low as 30%. Therefore, we propose a sample size of 500 patients, accounting for a 5% attrition rate (including motion artifacts limiting image quality and loss to follow-up).

To address potential confounding factors, multivariable logistic regression models will be employed for all primary and secondary analyses. These models will adjust for baseline demographic (age, sex), clinical (NIHSS score and comorbidities), and imaging factors (e.g., e-ASPECTS, infarct core volume, and mismatch ratio) that are known to influence outcomes. Variables will be selected based on prior evidence and clinical relevance.

For outcomes involving imaging endpoints, we will use linear regression models to assess continuous variables such as infarct volume size, incorporating key baseline predictors including CTP parameters, time from symptom onset to imaging, and recanalization status. We will also perform subgroup analyses to evaluate the effect of treatment modalities (IVT alone, EVT alone, or combined therapy).

For center-specific variability, random-effect models will be applied to account for clustering effects introduced by site-level differences. This approach will ensure that our results are not disproportionately influenced by outliers or biases from individual centers.

Sensitivity analyses will be performed to test the robustness of our findings, including adjustments for missing data using multiple imputation methods and re-analysis using alternative statistical techniques (e.g., propensity score matching for baseline NIHSS and time to treatment).

Finally, model diagnostics, such as variance inflation factors, will be used to check for multicollinearity among predictor variables, and residual plots will be examined for violations of model assumptions.

## 3. Discussion

Computed tomography perfusion (CTP) has become an essential imaging technique for assessing the ischemic penumbra during interventions such as IVT, typically performed between 4.5, and 9 h after stroke onset, and MT, performed between 6 and 27 h after onset [10,32]. Previous studies have demonstrated that the routine use of multimodal CT brain imaging, including CTP, does not significantly increase the door-to-needle time (DNT), thus allowing for timely therapeutic interventions without undue delay [14].

However, the full clinical utility of routine multimodal brain CT in improving stroke diagnosis before treatment remains under investigation. It is still unclear whether the findings from an acute multimodal CT examination, including CTP, can reliably predict the extent of the final ischemic brain infarct and the associated clinical outcomes. To address these gaps in knowledge, we are conducting a multicenter study involving over 2000 patients, collecting comprehensive clinical and radiological data. We anticipate that our study will provide valuable insights into the role of multimodal brain CT in refining stroke diagnosis and its predictive potential for patient outcomes.

Limitations of the study: The centers participating in the study use only one software (Brainomix Ltd., Oxford, UK). This fact may lead to the results not being fully generalizable to hospitals that employ other software for CTP evaluation.

## 4. Conclusions

The integration of the e-STROKE system, which utilizes artificial intelligence, presents a significant advancement in enhancing the speed and precision of stroke diagnosis. By providing immediate access to diagnostic results via modern platforms, e-STROKE streamlines interdisciplinary medical communication and fosters the implementation of evidence-based practices. This innovative approach is anticipated to improve patient outcomes by ensuring clinical interventions are closely aligned with current guidelines. Moreover, the findings from this study are expected to enhance the understanding and application of diagnostic and prognostic radiological markers in the management of acute stroke patients.

## Data Availability

The project is currently ongoing, and the data collected will be presented at major conferences and published in high-impact journals. At this time, the data are not publicly available.
